# Severe Lactic Acidosis Due to Inappropriate Use of Biktarvy in a Patient With AIDS

**DOI:** 10.7759/cureus.62604

**Published:** 2024-06-18

**Authors:** Andrew Strike, Gabriel Velez Oquendo, Sarika Chowdry, Gurleen Kaur

**Affiliations:** 1 Internal Medicine, Northeast Georgia Medical Center Gainesville, Gainesville, USA; 2 Family Medicine, Northeast Georgia Medical Center Gainesville, Gainesville, USA

**Keywords:** sepsis, icu, mitochondrial impairment, hiv, lactic acidosis, biktarvy

## Abstract

Bictegravir-emtricitabine-tenofovir alafenamide is an approved medication for the treatment of acquired immunodeficiency syndrome (AIDS). This medication, also called Biktarvy, includes an integrase strand inhibitor combined with nucleoside reverse transcriptase inhibitors (NRTIs) to prevent viral DNA synthesis and lead to improvements in disease progression and mortality in patients with AIDS. A rare but previously documented adverse effect of NRTIs present in Biktarvy is lactic acidosis. NRTIs can cause lactic acidosis through mitochondrial impairment, as mitochondria depend on DNA polymerase gamma for replication. This enzyme is very similar to HIV’s reverse transcriptase. Inhibition of mitochondrial production results in increased anaerobic metabolism and lactic acid production. We present a case where an inappropriately high dosage of Biktarvy in a patient with septic shock led to persistent lactic acidosis despite clinical improvement. After a thorough medication review, Biktarvy was temporarily held, and the lactic acidosis resolved. This clinical presentation stresses the importance of maintaining wide differentials for lactic acidosis and thorough medication reconciliation.

## Introduction

Bictegravir-emtricitabine-tenofovir alafenamide (also called Biktarvy) was approved in 2017 for the treatment of acquired immunodeficiency syndrome (AIDS) [1]. The medication has shown a marked reduction in the rate of illness and death in patients with AIDS [1]. The medication regimen includes an integrase strand inhibitor, which prevents viral DNA from being added to the host cell genome and inhibits human immunodeficiency virus (HIV) replication. The addition of nucleoside reverse transcriptase inhibitors (NRTIs) allows the prevention of viral DNA synthesis by mimicking natural nucleotides and progressing to premature chain termination during viral transcription [2].

## Case presentation

A 67-year-old male with a history of HIV on Biktarvy, paraplegia due to a motor vehicle accident, chronic kidney disease stage 3a, and chronic suprapubic catheter placement presented to the hospital with generalized weakness and acute encephalopathy. He had been discharged home five days prior, following an extended rehabilitation stay. On admission, he was found to be hypotensive with a blood pressure of 85/50 mmHg despite aggressive fluid resuscitation and hypothermic with a temperature of 95.9°F (35.5°C). Laboratory findings revealed severe metabolic acidosis with a pH of 6.9, bicarbonate less than 10 mmol/L, and a lactic acid level of 25 mmol/L. A complete blood count showed neutrophilic leukocytosis with a white blood cell count of 20x10^9^/L, while a comprehensive metabolic panel revealed hyperkalemia with a potassium level of 5.7 mmol/L and acute kidney injury with a creatinine level of 4.0 mg/dL. His most recent CD4 count showed improvement since starting on Biktarvy months prior (Table 1).

**Table 1:** Laboratory tests taken at admission compared to four months prior, with noted improvement after starting Biktarvy. HIV: human immunodeficiency virus.

Laboratory tests	Current admission	Four months prior
HIV status	Positive	Positive
Viral load	Undetectable	48,104 copies/μL
CD3 (total T cells)	1314 cells/μL	887 cells/μL
CD3%	90%	89%
CD4 (helper T cells)	564 cells/μL	252 cells/μL
CD4%	39%	25%
CD8 (suppressor cells)	733 cells/μL	636 cells/μL
CD8%	50%	64%

He was admitted to the ICU for suspected septic shock and was started on broad-spectrum antibiotics for a suspected urinary source of infection. During the hospital course, the patient suffered from worsening renal failure with anuria and persistent lactic acidosis, necessitating continuous renal replacement therapy (CRRT). Urine cultures were positive for *Pseudomonas*, and he completed a course of IV cefepime. After appropriate treatment, the patient showed dramatic clinical improvement, and his encephalopathy resolved. Despite clinical improvement and stabilization of hemodynamics, the lactic acid levels remained elevated at nearly 10 mmol/L. This lactic acid elevation persisted despite CRRT for acute renal failure.

During a review of the admission, it was discovered that the patient had been taking Biktarvy twice daily instead of the prescribed once-daily dose. The inappropriate dosing continued while he was in the ICU. This medication error was identified as a potential cause of persistent lactic acidosis. Biktarvy was temporarily withheld while the patient continued CRRT, leading to notable improvement in both renal function and lactic acid levels. He was eventually transferred out of the ICU and required hemodialysis until his renal function improved.

Although the inappropriate use of Biktarvy was associated with the patient's clinical presentation, it was determined that the correct use of the medication would lead to better outcomes compared to outright discontinuation. Therefore, Biktarvy was resumed, and the patient's lactic acid levels were monitored to ensure stability. After the patient’s lactic acidosis resolved, he was eventually discharged from the hospital with recommendations to follow up with the Infectious Disease team and take his medications at the recommended doses.

## Discussion

Our case presents an uncommon presentation of severe lactic acidosis in the setting of inappropriately high dosing of Biktarvy in a patient suffering from AIDS. NRTIs can cause overproduction of lactic acid through mitochondrial impairment [3]. Mitochondria depend on the enzyme DNA polymerase gamma (pol gamma) for replication. Curiously, this enzyme is structurally similar to the reverse transcriptase enzyme utilized by the HIV. Because of this similarity, NRTIs can reduce mitochondrial replication [3]. The decreased mitochondrial production results in a reduction of oxidative phosphorylation and the production of ATP. Due to persistent metabolic requirements, anaerobic metabolism is increased, and lactic acid is formed as a byproduct. Of note, this type of lactic acidosis is distinct from the subset associated with sepsis. In sepsis, systemic vasodilation and increased vascular permeability lead to decreased perfusion of tissues. Reduced tissue perfusion and a lack of oxygen delivery lead to increased anaerobic metabolism and lactic acid production [4].

There have been previously documented cases of antiretroviral medication use resulting in lactic acidosis. In these cases, several factors have been shown to increase the risk of NRTI-induced lactic acidosis. These risk factors include long-term usage of NRTIs, rapid weight gain or obesity, female gender, underlying liver disease, and co-administration of other medications that result in lactic acidosis [5]. Patients with reduced creatinine clearance are also at increased risk [6]. Table 2 highlights multiple common findings in NRTI-induced lactic acidosis. In these patients, symptoms are often generalized and nonspecific. Symptoms often include nausea and vomiting, abdominal pain, malaise, and fatigue [7]. Because of this, thorough history-taking and medication review are vital for adequate differential diagnoses in lactic acidosis.

**Table 2:** Common findings in NRTI-induced lactic acidosis [6]. NRTI: nucleoside reverse transcriptase inhibitor; LDH: lactate dehydrogenase.

Common findings in NRTI-induced lactic acidosis
Generalized weakness
Exhaustion
Muscle aches
Stomach ache
Nausea and vomiting
Weight reduction
Loss of appetite
Fever or low body temperature
Laboratory results
High lactate levels
Acid-base imbalance
Liver enzyme elevation
Reduced clotting factors
Increased LDH

In our case, the patient was admitted with generalized symptoms of weakness and confusion, along with meeting criteria for septic shock with a markedly high lactic acid level. The patient had two etiologies for the lactic acid elevation, both in the setting of tissue hypoperfusion and medication-induced mitochondrial inhibition. As the patient’s sepsis resolved, lactic acid showed a downtrend but remained persistently elevated due to inappropriate Biktarvy use. Multiple cases of lactic acidosis from antiretroviral therapy have been documented, with some cases being fatal [8]. While agents like zidovudine, lamivudine, didanosine, and other agents not found in Biktarvy are more strongly associated with this adverse event, all NRTIs have the potential to cause mitochondrial inhibition [8,9]. There have been similar cases where inappropriate dosing or overdose of NRTI therapy has led to severe lactic acidosis, especially in the setting of renal impairment and concomitant use of other nephrotoxic agents [10]. This mirrors our case; the patient experienced acute renal failure in the setting of septic shock and required CRRT while in the ICU. The association between the risk of lactic acidosis in septic patients taking NRTIs has been discussed, and the risks versus benefits of holding NRTIs in sepsis should be a focus of future research [10]. Biktarvy is currently used in patients with end-stage renal disease due to its bioavailability despite hemodialysis, with recommendations for once-daily dosing and adequate viral suppression regardless of dialysis schedules [11]. This is likely due to the high protein binding affinity of the medications in the regimen [11]. Despite CRRT, our patient's inappropriate dosing of Biktarvy still led to toxicity.

## Conclusions

This case shows the importance of accurate medication reconciliation for patients with complex medical conditions while in the ICU. The severe lactic acidosis seen in this patient likely resulted from a combination of septic shock and decreased mitochondrial production from inappropriately high Biktarvy dosing. Ultimately, addressing the medication error led to the resolution of lactic acidosis and discharge from the hospital. This case serves as a reminder of the importance of medication reconciliation and maintaining a wide differential diagnosis in the ICU. We encourage further research comparing the risks and benefits of continuing NRTI therapy in patients admitted to the ICU. 
